# ATP measurement as an objective method to measure environmental contamination in 9 hospitals in the Dutch/Belgian border area

**DOI:** 10.1186/s13756-020-00730-9

**Published:** 2020-05-28

**Authors:** Andreas van Arkel, Ina Willemsen, Linda Kilsdonk-Bode, Sindy Vlamings-Wagenaars, Anne van Oudheusden, Pascal De Waegemaeker, Isabel Leroux-Roels, Martine Verelst, Evelien Maas, Anita van Oosten, Patricia Willemse, Esther van Asselen, Ella Klomp-Berens, Karen Franssen, Elise Van Cauwenberg, Jan Kluytmans, on behalf of the i-4-1-Health Study Group, Lieke van Alphen, Nicole van den Braak, Caroline Broucke, Anton Buiting, Liselotte Coorevits, Sara Dequeker, Jeroen Dewulf, Wouter Dhaeze, Bram Diederen, Helen Ewalts, Herman Goossens, Inge Gyssens, Casper den Heijer, Christian Hoebe, Casper Jamin, Patricia Jansingh, Jan Kluytmans, Marjolein Kluytmans-van den Bergh, Stefanie van Koeveringe, Sien De Koster, Christine Lammens, Isabel Leroux-Roels, Hanna Masson, Ellen Nieuwkoop, Anita van Oosten, Natascha Perales Selva, Merel Postma, Stijn Raven, Veroniek Saegeman, Paul Savelkoul, Annette Schuermans, Nathalie Sleeckx, Krista van der Slikke, Arjan Stegeman, Tijs Tobias, Paulien Tolsma, Jacobien Veenemans, Dewi van der Vegt, Martine Verelst, Carlo Verhulst, Pascal De Waegemaeker, Veronica Weterings, Clementine Wijkmans, Patricia Willemse-Smits, Ina Willemsen

**Affiliations:** 1 Department of Infection Control, Amphia Hospital, Breda, The Netherlands; 2 Laboratory for Microbiology and Infection Control, Elisabeth TweeSteden Hospital, Tilburg, The Netherlands; 3 Department of Infection Control, Elisabeth Tweesteden Hospital, Tilburg, The Netherlands; 4 Department of Infection Control, Ghent University Hospital, Ghent, Belgium; 5 Laboratory for Microbiology, Ghent University Hospital, Ghent, Belgium; 6 Department of Diagnostic Sciences, University hospital Ghent, Ghent, Belgium; 7 Department of Infection Control, University Hospitals Leuven, Leuven, Belgium; 8 Department of Infection Control, ZorgSaam Hospital, Terneuzen, The Netherlands; 9 Department of Infection Control, Admiraal de Ruyter Hospital, Goes, The Netherlands; 10 Department of Infection Control, Elkerliek Hospital, Helmond, The Netherlands; 11 Department of Infection Control, Maastricht University Medical Center+, Maastricht, The Netherlands; 12 Department of Infection Control, Antwerp University Hospital, Antwerp, Belgium; 13 Microvida Laboratory for Microbiology, Amphia Hospital, Breda, The Netherlands; 14 Julius Center for Health Sciences and Primary Care, UMC Utrecht, Utrecht University, Utrecht, The Netherlands

**Keywords:** ATP measurement, Fomite, Surface contamination, Cleaning

## Abstract

**Background:**

The objective of this study was to determine the level of environmental contamination in hospitals in the Dutch/Belgian border area, using ATP measurements.

**Design:**

A cross-sectional observational survey.

**Methods:**

Standardized ATP measurements were conducted in 9 hospitals on 32 hospital wards. Thirty pre-defined surfaces per hospital ward were measured with the 3 M Clean Trace NG luminometer. Results are displayed in relative light units (RLU). RLU > 1000 was considered as “not clean.” Differences in RLU values were compared between countries, hospitals, fomite groups and medical specialties.

**Results:**

A total of 960 ATP measurements were performed, ranging from 60 up to 120 per hospital. The median RLU-value was 568 (range: 3–277,586) and 37.7% of the measurements were rated as not clean (RLU > 1000). There were significant differences between countries, hospitals and fomite groups.

**Conclusion:**

ATP measurements can be used as a more objective approach to determine the level of environmental contamination in hospitals. Significant differences in ATP levels were found between hospitals and between countries. Also, substantial differences were found between different fomite groups. These findings offer potential targets for improvement of cleanliness in healthcare facilities.

## Background

Contaminated surfaces and fomites are considered an important reservoir of (multi-resistant) microorganisms in hospitals [1–3]. Therefore, cleaning of the environment is important for reducing bacterial spread, controlling antimicrobial resistance and improving patient safety.

The assessment of the cleanliness of surfaces in hospitals is mostly conducted by visual inspection. This method is not sensitive and subjective and therefore unreliable [4–6]. Recently, a more objective technique was introduced to measure biological contamination. This technique is based on the measurement of adenosine triphosphate (ATP), a molecule that is present in all organic cells. The amount of ATP measured is expressed in relative light units (RLU) using the 3 M Clean Trace NG luminometer: the higher the amount of ATP measured, the higher the RLU value will be. ATP measurement seems a promising alternative to visual inspection and aerobic colony count cultures [7].

The aim of this study was to determine the level of environmental contamination in hospitals in the Dutch/Belgian border area as a part of the cross border One Health project that aimed to control the spread of antimicrobial resistance (the i-4-1-Health project). Within this project ATP measurements were performed to examine if ATP measurement is a valid method to measure environmental contamination. Furthermore, the aim of the i-4-1-Health project was to visualize the differences in environmental contamination between hospitals and countries. Differences between countries, hospitals, fomite groups and medical specialties were investigated and visualized.

## Methods and materials

### Setting

As part of a multicenter One Health project in the Dutch/Belgian border area, the i-4-1-Health project, standardized ATP measurements were conducted in 9 hospitals (3 Belgian university hospitals, 1 Dutch university hospital, 3 Dutch teaching hospitals and 2 Dutch general hospitals). The ATP measurements were conducted on different hospital wards, from 2 up to 4 wards per hospital, depending on the hospital size. In each hospital, ATP measurements were conducted on at least a surgical ward and an internal medicine ward. When ATP measurements were conducted on more than 2 wards a selection was made from the medical specialties urology, cardiology, orthopedic surgery, pulmonology and/or geriatrics. On each ward, ATP measurements were performed on 30 pre-defined fomites (Table 1). These fomites were classified into 4 different groups: medical devices, patient bound materials, sanitary items and ward bound materials. Fomites were chosen based on the following criteria: frequently touched by nursing staff or frequently touched by patients or in the direct vicinity of patients or high-risk surfaces (e.g. tabletop for medication preparation).

**Table 1:** Overview of the fomites measured per hospital ward

Fomite	
Blood pressure meter - control panel	**Medical devices**
Thermometer	
Glucose meter - control panel	
Glucose meter - insertion opening	
Infusion stand ×3	
Stethoscope - membrane	
Infusion pump - control panel ×2	
Pull-up bracket	Patient bound materials
Nightstand - pullout tabletop	
Bedrails	
Paging system at bed ×2	
Toilet - seat	Sanitary items
Toilet - bowl	
Toilet - flush button	
Toilet - support/bracket	
Toilet chair - seat	
Bedpan cleaner - control panel	
Sink - faucet operation ×2	
Shower - support/bracket	
Shower - showerhead	
Keyboard - Computer On Wheels (COW)	**Ward bound materials**
Keyboard - team post	
Tabletop medication preparation	
Telephone - keys	
Chair - seat	

### ATP measurements

The Clean-Trace NG Luminometer (3 M, Zoeterwoude, the Netherlands) was used for the ATP measurements, results were reported in RLU. ATP measurements were conducted by trained researchers working at the department of infection control of the corresponding hospital. Two methods of measurement were performed. Method A: a surface of approximately 100 cm^2^ (10 × 10 cm) is thoroughly swabbed in two directions with an ATP-swab. Method B: the whole surface is thoroughly swabbed with an ATP-swab, and the 100 cm^2^ surface is approached as best as possible. Method B was used for fomites that did not have a flat surface, an easily measureable surface or that have a surface smaller than 100 cm^2^. The manufacturer’s instructions on conducting the ATP measurements were followed. Each researcher got instructed to perform the ATP measurements around noon. Instruction was given to not perform ATP measurement directly after cleaning.

### RLU breakpoints

RLU breakpoints were defined by identifying frequently touched surfaces by nursing staff and patients. Measurements were conducted on 7 wards; per ward 20 fomites were measured at a random point during the day. Based on these measurements, with consultation of microbiologists and infection control practitioners from multiple Dutch and Belgian hospitals, RLU breakpoints were defined. These breakpoints were developed for the IRIS scan [8]. With RLU < 1000 as the breakpoint for “cleanliness,” the outcome of the IRIS scan would be applicable in practice. The following breakpoints were chosen: clean (RLU < 1000), intermediate (RLU ≥1000 to < 3000) and dirty (RLU ≥3000). For these breakpoints color codes were used to visualize the level of contamination (respectively green, orange and red).

### Statistical methods

All data were analyzed with Statistical Package for Social Science software (SPSS; IBM Corp., Armonk, New York, US; version 25). Differences in the distribution of RLU values between hospitals and fomite groups were calculated using the Kruskall Wallis test, adjustment for multiple testing was performed. Overall difference between both countries and medical specialties was calculated using the Mann-Whitney U test. Because of the large differences in number of measurements per medical specialty, two groups were formed: surgical and non-surgical specialties. Statistical significance was accepted at *p* < 0.05 after correction for multiple testing. Relative Risks (RRs) for the more frequent occurrence of “not clean” fomites were calculated with univariable and multivariable generalized linear models (GLM) with a binomial distribution. In the multivariate analysis the model was corrected for medical specialty and surface category. The hospital with the lowest percentage of “not clean” surfaces was selected as reference.

## Results

In total 960 ATP measurements were performed, 30 ATP measurements per ward, accounting for 60 up to 120 ATP measurements per hospital. The median RLU-value was 568 with a range from 3 up to 277,586. Of all measurements 37.7% (362/960) were considered as “not clean” (RLU > 1000) and 16.6% (159/960) had RLU values above 3000 (‘dirty’).

**Fig. 1:**
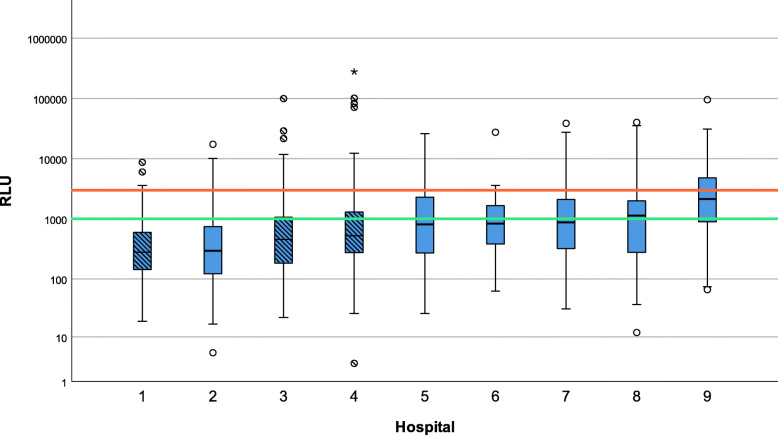
Boxplot of RLU values between hospitals with RLU breakpoints. Legend: Belgian hospitals are shaded. Outliers are marked with a circle, extreme outliers with a star. RLU breakpoints are marked by colored lines

Figure 1 shows the differences in median RLU-values between the 9 hospitals in both countries. The *p*-values of the pairwise comparison of hospitals are visualized in Table 2. Significant differences are highlighted. The median RLU-value per hospital from high to low was: 2137, 1131, 872, 835, 807, 524, 455, 294, 278. Hospital 1 had significantly lower ATP levels than all other hospitals apart from hospital 2 and 3. On the other hand, hospital 9 had significantly higher values than all other hospitals. The median RLU-value measured in the Netherlands was 793, the median RLU-value measured in Belgium was 431. The difference in RLU distribution between the two countries was significant (*p* < 0.001).

**Table 2:** Matrix of *p*-values of pairwise comparisons between different hospitals

	***Hospital 1***	***Hospital 2***	***Hospital 3***	***Hospital 4***	***Hospital 5***	***Hospital 6***	***Hospital 7***	***Hospital 8***	***Hospital 9***
***Hospital 1***		***1.000***	***0.093***	0.002*	< 0.001*	0.001*	< 0.001*	< 0.001*	< 0.001*
***Hospital 2***	***1.000***		***0.263***	0.007*	< 0.001*	0.002*	< 0.001*	< 0.001*	< 0.001*
***Hospital 3***	***0.093***	***0.263***		***1.000***	***1.000***	***1.000***	***0.254***	***0.082***	< 0.001*
***Hospital 4***	0.002*	0.007*	***1.000***		***1.000***	***1.000***	***1.000***	***1.000***	< 0.001*
***Hospital 5***	< 0.001*	< 0.001*	***1.000***	***1.000***		***1.000***	***1.000***	***1.000***	0.013*
***Hospital 6***	0.001*	0.002*	***1.000***	***1.000***	***1.000***		***1.000***	***1.000***	0.020*
***Hospital 7***	< 0.001*	< 0.001*	***0.254***	***1.000***	***1.000***	***1.000***		***1.000***	0.010*
***Hospital 8***	< 0.001*	< 0.001*	***0.082***	***1.000***	***1.000***	***1.000***	***1.000***		0.029*
***Hospital 9***	< 0.001*	< 0.001*	< 0.001*	< 0.001*	0.013*	0.020*	0.010*	0.029*	

*indicates a statistically significant difference (*p* < 0.05)

Per fomite group 160 to 320 ATP measurements were conducted: 320 ATP measurements in the medical devices group, 320 ATP measurements in the sanitary items group, 160 ATP measurements in the patient bound materials group and 160 ATP measurements in the ward bound materials group.

**Fig. 2:**
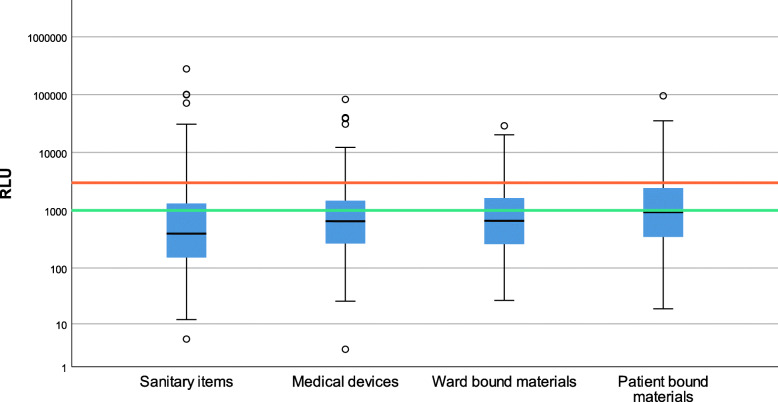
Boxplot of RLU values between fomite groups with RLU breakpoints. Legend: Outliers are marked with a circle. RLU breakpoints are marked by colored lines

The differences in median RLU-value between the different fomite groups are visualized in Fig. 2. The pairwise comparisons of the fomite groups are visualized in Table 3, significant differences are highlighted. The median RLU-value was 931 in the patient bound materials group, 659 in ward bound materials, 651 in medical devices, and 396 in sanitary items. Sanitary items had significantly lower values than all other groups of fomites.

**Table 3:** Matrix of *p*-values of pairwise comparisons between different categories of fomites

	***Patient bound materials***	***Medical devices***	***Ward bound materials***	***Sanitary items***
***Patient bound materials***		***0.087***	***0.487***	< 0.001*
***Medical devices***	***0.087***		***1.000***	0.006*
***Ward bound materials***	***0.487***	***1.000***		0.011*
***Sanitary items***	< 0.001*	0.006*	0.011*	

*indicates a statistically significant difference (*p* < 0.05)

Per medical specialty 30 to 270 ATP measurements were conducted. The surgical group consisted out of 450 measurements, the non-surgical group out of 510 measurements. The median RLU-value measured in the surgical group was 626, the median RLU-value measured in the non-surgical groups was 545. The difference in RLU distribution between the two was not significant (*p* > 0.05).

Univariate predictors for the more frequent occurrence of “not clean” surfaces are visualized in Table 4, significant differences are highlighted.

**Table 4:** Univariate and multivariate analysis per group with display of percentages of “not clean” (RLU > 1000) items. In the multivariate analysis, the model was adjusted for medical specialty and surface category

		Univariate		Multivariate	
	> 1000 RLU (%)	***P***	RR (95% BI)	***P***	RR (95% BI)
*hospital*					
hospital 1	15.8	Ref			
hospital 2	19.2	0.498	1.21 (0.70–2.10)	0.473	1.25 (0.70–2.13)
hospital 3	30.0	**0.011**	**1.89 (1.15–3.11)**	**0.011**	**1.90 (1.16–3.11)**
hospital 4	35.8	**0.001**	**2.26 (1.40–3.65)**	**0.001**	**2.28 (1.38–3.58)**
hospital 5	47.5	**< 0.001**	**3.00 (1.91–4.72)**	**< 0.001**	**2.95 (1.88–4.64)**
hospital 6	40.0	**< 0.001**	**2.53 (1.51–4.23)**	**0.001**	**3.37 (2.14–5.21)**
hospital 7	45.0	**< 0.001**	**2.84 (1.80–4.49)**	**< 0.001**	**2.44 (1.45–4.08)**
hospital 8	52.5	**< 0.001**	**3.32 (2.12–5.18)**	**< 0.001**	**2.78 (1.76–4.39)**
hospital 9	71.7	**< 0.001**	**4.53 (2.91–7.04)**	**< 0.001**	**4.38 (2.82–6.80)**
*medical specialty*					
surgical	40.7	Ref			
non-surgical	35.1	0.076	0.86 (0.73–1.01)	0.241	0.91 (0.78–1.06)
*surface category*					
sanitary items	29.4	Ref			
Patient bound materials	47.5	**< 0.001**	**1.62 (1.28–2.05)**	**< 0.001**	**1.57 (1.27–1.93)**
ward bound materials	40.6	**0.010**	**1.39 (1.08–1.78)**	**0.008**	**1.37 (1.09–1.72)**
medical devices	38.4	**0.016**	**1.30 (1.05–1.62)**	**0.014**	**1.30 (1.05–1.60)**

Significant differences in bold (*p* < 0.05)

Multivariate predictors for a higher chance of a non-clean surface were hospital 3 until 9 and all fomite groups with sanitary items as reference.

## Discussion

Hospital cleanliness is an important factor to reduce bacterial spread and therefore prevent hospital infections [1–3]. Measuring hospital cleanliness can be time consuming and judging surface contamination by visual assessment alone is an unreliable indicator of the level of environmental contamination [4–6]. ATP measurements seem a promising alternative to visual assessments by quantifying the amount of organic matter on a surface in objective and reproducible way. The results are available practically on the spot and with the cut offs that we defined before the project started the RLU’s are easy to understand for the users [9].

Nevertheless, there is still an ongoing discussion if ATP measurements are suitable to quantify the level of bacterial contamination of a surface. This because ATP reflects the amount of all organic material and not only bacteria [10]. The correlation between RLU values and microbial contamination differs between studies and ATP measurement may not be used to examine bioburden or sterility of a surface [11]. Critics argue that a relatively low level of ATP solely based on the presence of bacteria only, may carry a relatively high risk to patients. This is without doubt a valid argument. On the other hand, the amount of organic material does reflect the level of environmental contamination and therefore can be used as a surrogate marker to measure the effectiveness of cleaning. In addition, organic material may serve as a nutritional source for bacteria and thereby promote bacterial growth. As an example, vancomycin resistant enterococci (VRE) are frequently found on inanimate surfaces which are shown to be a reservoir and a cause of spread of VRE in the hospital environment [12, 13]. Thus by identifying dirty surfaces with ATP measurements, it may be possible to reduce spread of VRE or other multi resistant bacteria [1]. We consider the ATP measurement as a useful tool to measure the level of environmental contamination in a reliable and reproducible way. Thereby they can be used to benchmark hospitals or wards and improve the cleanliness of hospitals.

We defined RLU thresholds, based on literature review and on previous ATP measurements in the participating hospitals before the project started [8]. Other studies have recommended an RLU threshold for cleanliness at 250–500 RLU, however this threshold is intended for measurement (almost) directly after cleaning [4, 6, 8, 14–16]. We developed ATP thresholds for conducting an ATP measurement at a random point in time on a hospital ward, not knowing if items were used or cleaned that day. The goal of this study was to visualize the environmental contamination independent from the time of cleaning and to determine the environmental contamination to which a patient is exposed in the hospital. The hospital cleaning protocols were not monitored as part of this study. The main goal was to use the defined RLU breakpoints for insight in surface contamination for improvement of cleaning in a later phase of the project. The results from the ATP measurements will be fed back to the corresponding hospitals. Depending on the result of the ATP measurements, targeted cleaning improvement actions will be implemented in each hospital. The effects of this feedback will be measured in a second round of ATP measurement. A group of experts (experienced infection control practitioners and microbiologists) defined the thresholds.

There are several ways to analyze the ATP results. Firstly, by comparing the median RLU and distribution of the findings. Another method is to categorize the results with a breakpoint for cleanliness (RLU < 1000). By using the former method, insight is provided into the distribution of the RLU values, and thus the degree of contamination. The second method indicates how often a patient or healthcare worker is confronted with an “unclean” surface. The latter is probably more relevant in determining where risks exist, while the first is more suitable for comparing hospitals or departments.

We also performed analyses with an RLU threshold of < 500 and < 250 RLU (Table 5, supplement). This changes the results in the multivariate analysis between hospitals, where RR’s between hospitals are smaller. The differences between surface categories and medical specialties stay in the same range. The final conclusion of this research stays mostly the same.

There are some (potential) limitations of this study. First, different researchers measured fomites at different points in time. This can cause bias because a researcher could have his/her own method of sampling and for instance choose spots which look visually cleaner or dirtier. However, researchers were given a training and instructions on how to perform the measurements properly, according to manufacturer’s guidelines. Also, the researchers checked and validated each other before the project was started. Instructions were given on how to swab each fomite. Also, researchers were given instructions to perform the ATP measurements early in the afternoon to standardize the timing.

Even so there are still some potential limitations bound to ATP measurement. Firstly, ATP measurement is (still) a quite expensive method for determining surface contamination, compared to other methods. Secondly, there is a propensity to false-positive results when certain disinfecting agents have been used to clean a surface. Also no pathogen can be identified with ATP measurement [17]. These factors should be given consideration before performing ATP measurements.

The main advantages of ATP measurements are the objective and reproducible results, which are produced on the spot so provide immediate feedback. ATP measurements give a quantitative result, which is easy to interpret by nursing or cleaning staff when thresholds for clean and not clean are defined. In comparison, aerobic colony counts give an indication of the number of viable bacteria. This may be considered more relevant but the major disadvantage is that the results take 24-48 h to become available to those who can improve the cleaning process. This makes it less attractive for quality improvement using rapid feedback to the users.

ATP measurements can be used as a fast and objective approach to determine the level of environmental contamination in hospitals. The substantial and significant differences between countries, hospitals and fomite groups provide a basis for improvement. Further research in cleaning regimes is needed to explain the differences between the hospitals. Subsequent changes in the cleaning policy can be judged for their effectiveness using repeated ATP measurements.

## Conclusion

Within this study significant differences in environmental contamination were found between countries, hospitals and fomite groups. In addition a high percentage of “not clean” (> 1000 RLU) surfaces or fomites was found.

In all hospitals there is room for improvement, but this varies considerably between hospitals. After adjusting for medical specialty and fomite group the relative risk for finding a “not clean” surface in hospital 9 was 4.4 times higher than in hospital 1. We found a high level of variation of “not clean” surfaces between groups (e.g. hospitals, fomite groups). These results can be used to improve cleanliness by defining best practices and implementing them. For instance, by analyzing cleaning regimes (cleaning method, cleaning staff, products used for cleaning and disinfection, standard disinfection during hospital stay and/or after discharge, etc.) in the hospitals with a lower level of environmental contamination can help to improve cleaning regimes in hospitals with higher levels of environmental contamination. Also, by analyzing different fomites and fomite groups, cleaning can be improved by focusing on the most contaminated fomites.

## Supplementary information

**Additional file 1: Table 5**. Univariate and multivariate analysis per group, with < 250 RLU and < 500 RLU breakpoints. In the multivariate analysis, the model was adjusted for medical specialty and surface category.

## Data Availability

As agreed within the i-4-1-Health consortium, the i-4-1-Health datasets will be made available no earlier than December 31st, 2020 and no later than December 31st, 2024, in accordance with the FAIR (Findable, Accessible, Interoperable and Reusable) data principles [18].

## Abbreviations

ATP: Adenosine triphosphate
RLU: Relative light units
RRs: Relative Risks
GLM: Multivariable generalized linear models
VRE: Vancomycin resistant enterococci
FAIR: Findable, Accessible, Interoperable and Reusable
ERDF: European Regional Development Fund
