# Supplementary material for: ATP measurement as an objective method to measure environmental contamination in 9 hospitals in the Dutch/Belgian border area
Source: Antimicrob Resist Infect Control. 2020 May 28;9:77. doi: 10.1186/s13756-020-00730-9 (PMC7254657; doi:10.1186/s13756-020-00730-9)
Supplement: Supplementary file 1 — Additional file 1: Table 5. Univariate and multivariate analysis per group, with < 250 RLU and < 500 RLU breakpoints. In the multivariate analysis, the model was adjusted for medical specialty and surface category. [file 13756_2020_730_MOESM1_ESM.docx]

| **RLU <250 RLU <500** | | | | | | | | | |
| --- | --- | --- | --- | --- | --- | --- | --- | --- | --- |
|  | **Univariate** | |  | **Multivariate** | |  | **Univariate** |  | **Multivariate** |
|  | ***P*** | **RR (95% BI)** |  | ***P*** | **RR (95% BI)** | ***P*** | **RR (95% BI)** | ***P*** | **RR (95% BI)** |
| *hospital* |  |  |  |  |  |  |  |  |  |
| hospital 1 | Ref |  |  |  |  | Ref |  |  |  |
| hospital 2 | **0.004** | **1.28 (1.08 – 1.51)** |  | **0.007** | **1.25 (1.06 – 1.48)** | **0.010** | **1.39 (1.08 – 1.79)** | **0.011** | **1.38 (1.07 – 1.77)** |
| hospital 3 | 0.132 | 1.14 (0.96 – 1.36) |  | 0.119 | 1.15 (0.96 – 1.37) | 0.281 | 1.16 (0.89 – 1.52) | 0.257 | 1.17 (0.89 – 1.52) |
| hospital 4 | 0.108 | 0.85 (0.69 – 1.04) |  | 0.095 | 0.84 (0.69 – 1.03) | 0.187 | 0.81 (0.60 – 1.10) | 0.234 | 0.83 (0.61 – 1.13) |
| hospital 5 | **<0.001** | **1.57 (1.35 – 1.81)** |  | **<0.001** | **1.54 (1.33 – 1.77)** | **<0.001** | **2.03 (1.63 – 2.52)** | **<0.001** | **2.01 (1.62 – 2.50)** |
| hospital 6 | 0.751 | 1.03 (0.86 – 1.24) |  | 0.809 | 1.02 (0.85 – 1.23) | 0.112 | 1.24 (0.95 – 1.61) | 0.102 | 1.24 (0.96 – 1.61) |
| hospital 7 | **0.008** | **1.30 (1.07 – 1.57)** |  | **0.016** | **1.26 (1.04 – 1.53)** | **0.002** | **1.56 (1.18 – 2.06)** | **0.002** | **1.55 (1.17 – 2.04)** |
| hospital 8 | **<0.001** | **1.38 (1.18 – 1.62)** |  | **<0.001** | **1.36 (1.16 – 1.60)** | **<0.001** | **1.62 (1.28 – 2.07)** | **<0.001** | **1.61 (1.26 – 2.04)** |
| hospital 9 | **<0.001** | **1.50 (1.50 – 2.04)** |  | **<0.001** | **1.79 (1.54 – 2.08)** | **<0.001** | **2.59 (2.07 – 3.25)** | **<0.001** | **2.62 (2.11 – 3.26)** |
| *medical specialty* |  |  |  |  |  |  |  |  |  |
| surgical | Ref |  |  |  |  | Ref |  |  |  |
| non-surgical | 0.140 | 0.95 (0.88 – 1.02) |  | 0.766 | 0.99 (0.93 – 1.06) | **0.006** | **0.87 (0.78 – 0.96)** | 0.087 | 0.92 (0.84 – 1.01) |
| *surface category* |  |  |  |  |  |  |  |  |  |
| sanitary items | Ref |  |  |  |  | Ref |  |  |  |
| Patient bound materials | **<0.001** | **1.28 (1.15 – 1.43)** |  | **<0.001** | **1.31 (1.18 – 1.45)** | **<0.001** | **1.44 (1.24 – 1.67)** | **<0.001** | **1.44 (1.26 – 1.64)** |
| ward bound  materials | **<0.001** | **1.31 (1.18 – 1.45)** |  | **<0.001** | **1.28 (1.16 – 1.41)** | **0.002** | **1.29 (1.09 – 1.49)** | **0.001** | **1.28 (1.10 – 1.49)** |
| medical devices | **<0.001** | **1.22 (1.11 – 1.35)** |  | **<0.001** | **1.21 (1.11 – 1.33)** | **0.001** | **1.25 (1.09 – 1.43)** | **<0.001** | **1.25 (1.03 – 1.42)** |
